# Spiritual Complementary and Alternative Medicine Practices, Health-Related Behaviors, and Mortality Among US Adults: A National Health Interview Survey-Based Cohort Study

**DOI:** 10.7759/cureus.112881

**Published:** 2026-07-17

**Authors:** Jim E Banta, Jiahao Peng, Elizabeth Johnston Taylor

**Affiliations:** 1 School of Public Health, Loma Linda University, Loma Linda, USA; 2 Department of Medicine, Montefiore Medical Center, Albert Einstein College of Medicine, New York, USA; 3 School of Nursing, Loma Linda University, Loma Linda, USA

**Keywords:** binge drinking, centers for disease control and prevention, meditation, mortality, prayer, religion and medicine, smoking, spiritual practices

## Abstract

Background

Relatively few studies have examined population-level impacts of spiritual practices on health. The National Health Interview Survey (NHIS) asks adults about spiritual practices for their own health in a complementary and alternative medicine (CAM) module roughly every five years. The Centers for Disease Control and Prevention (CDC) linked 1986 to 2018 surveys to the National Death Index.

Objective

Study objectives are to 1) compare spiritual practices as asked within CAM modules to mortality by the end of 2019, adjusting for respondent characteristics; 2) identify how health behaviors changed over time, particularly with regards to spiritual practices; and 3) explore significant interaction effects of spiritual practices in relation to mortality.

Methods

As three different spiritual practices were asked across 1999, 2002, 2007, 2012, and 2017, we ran survey-adjusted bivariate Chi-square tests of health behaviors by spiritual practice (yes/no) by grouping of years. Guided by the Andersen Behavioral Model, we included many socio-demographics, health status, and health behavior variables in a fully saturated, survey-adjusted Cox proportional hazards regression with mortality (yes/no) as the dependent variable and age at survey as the time variable. Due to top-coding at 85 years, analysis was limited to 18-84 years of age. A follow-up regression included interaction terms of spiritual practices with respondent characteristics. We also ran a sensitivity analysis to assess potential bias due to missing data.

Results

Among adults (n=137,291), 11.51% had died by the end of 2019. In 1999, an estimated 27.0 million adults (13.7% of adults that year) reported usingspiritual healing/prayer for their own health. In 2002 and 2007, an estimated 92.6 million adults annually (43.9% of adults) reported praying for your own health*. *In 2012 and 2017, an estimated 16.2 million (6.9% of adults) reported using spiritual meditation for own health. Diabetes and currently smoking were most strongly associated with mortality, hazard ratios of 2.35 and 2.10, respectively, p<0.0001. Praying for own health was associated with higher mortality risk, HR: 1.07 (95% confidence interval: 1.01-1.13, p=0.012), while the other practices were not significant. In the interaction analysis, prayer by itself was no longer significant, but there was a significant interaction between prayer and binge drinking (HR=1.21, 95% CI: 1.02-1.30, p=0.026). All three spiritual practices for own health were associated with not currently smoking and receiving flu vaccination in past year. Most spiritual practices, except prayer, were associated with having a dentist visit in prior year and being more likely to do any moderate exercise/vigorous exercise/strength training in past week. Most spiritual practices, except spiritual meditation, were associated with not binge drinking in past year. For example, any binge drinking was reported by 15.0% (95% CI: 14.4-15.7) of those with prayer for health compared to 24.4% (23.6-25.2) of others in 2002 and 2007. There were secular changes, with smoking being less prevalent in 2012 and 2017 compared to prior years while exercise was more common.

Conclusion

Millions of adults used spiritual practices for health and generally also had positive health-related behaviors. Spiritual practices were associated with mortality largely through health behaviors. Differences between spiritual practices and binge drinking deserve further study.

## Introduction

Although the number of United States residents who identify as non-religious has increased over time, a majority still identify as religious [1]. Furthermore, Americans often pray, sometimes about their health [2,3]. Religiosity and spirituality (the more universal, innate dimension of personhood that evokes religion) are associated with physical and psychological well-being [4]. After reviewing over 3,300 studies, Koenig found most studies were positive, linking various indicators of religiosity or spirituality (R/S) with outcomes such as longevity, endocrine function, perceived health, suicidality, mental health, and happiness [5]. A striking example of this body of evidence is prospective cohort study by Chen et al. showing that those attending religious services at least once a week had lower risk of all-cause mortality compared to those who never attended religious services [6]. Thus, religion is now considered to be a social determinant of health [7].

Religious and spiritual practices, experienced by religious and non-religious Americans, include prayer. Prayer is the most studied personal spiritual practice, and it may improve health via the placebo effect, a diversion away from one’s health problems, promoting supernatural interventions, activating latent energies, and encouraging the faithful to engage in health-related behaviors [8].

Frequency of prayer practices has been assessed by questions about complementary and alternative medicine (CAM) included in the National Health Interview Survey (NHIS) [9]. Although findings from the General Social Survey in 2024 indicated that Americans often pray (56.6% at least once a day; only 22.7% never) [2], NHIS findings suggest Americans pray less frequently about their health [3,9]. When looking at the 2007 NHIS adult data, Wachholtz and Sambamoorthi found that 49% reported having prayed for their health during the past year [3]. Another nationally representative survey documented that many Americans do pray for their own (79%) and others (87%) health at least some time during their life and ask others to pray for them (54%) [10]. Thus, praying about health is a common occurrence among Americans.

For those who pray, it is often believed to be beneficial [11,12]. There is mixed evidence regarding efficaciousness. For example, one study of adults with chronic illness found that over a six-year period, those who prayed every day were more likely to survive compared to those who did not [13]. A Cochrane review of experimental evidence about outcomes of intercessory prayer, however, concluded that it was “neither significantly beneficial nor harmful for those who are sick” [14]. This equivocal conclusion may be explained by the wide variation in prayer practices, or frequency of the practices, or perceived efficacy, or other psychological facets of praying.

Although type of prayer may contribute to health-related outcomes and explain discrepancies, there is merit to Cochrane reviewers’ call for stronger evidence on which to draw conclusions [14]. As shown above, a plethora of empirical studies suggest religiosity and spirituality are associated with diverse positive health outcomes among individuals. However, a question remains as to whether one can detect a population-level impact of prayer on health. Specifying such an impact is an important effort for the translational epidemiology of religion [15].

This analysis of National Health Interview Survey data sought to quantify associations between spiritual practices, which include spiritual meditation as well as personal prayer, for one’s health, health-related behaviors, and mortality. Research questions included: A) how are spiritual practices as asked within the CAM modules associated with mortality status, adjusting for respondent characteristics, particularly health status and health behaviors; B) how have health behaviors changed over time, particularly in relation to spiritual practices; and C) what are significant interactions between spiritual practices and respondent characteristics, particularly health behaviors, in relationship to mortality.

## Materials and methods

This observational study involved secondary analysis of de-identified, cross-sectional United States federal survey data. Due to the Centers for Disease Control and Prevention (CDC) adding mortality information years after survey completion, this may be considered a retrospective cohort study. All participants provided informed consent at the time of data collection. The present study was deemed exempt by the investigators’ university institutional review board (IRB# 5240487).

Study population

Data were obtained from the National Health Interview Survey (NHIS), a data collection program of the National Center for Health Statistics, Centers for Disease Control and Prevention (CDC). NHIS has been conducted since 1957 and is a cross-sectional, face-to-face, household interview survey, whose findings are representative of the civilian noninstitutionalized population residing within the United States [16]. NHIS records from 1986 to 2018 were linked by the CDC against the National Death Index. Thus, associations between NHIS variables and eventual time and cause of death can be determined. Public-use NHIS data files were obtained through the Integrated Public-Use Microdata Set (IPUMS) [17].

Data were selected from the years during which the NHIS included items regarding complementary and alternative medicine (CAM) use: 1999, 2002, 2007, 2012, and 2017. This generated an initial sample of 146,505 adults at least 18 years of age. However, 5,572 adults were not successfully matched against the National Death Index and therefore excluded from analysis. Fortunately, CDC provided sampling weight which adjusted for non-matching. Furthermore, 3,642 respondents had been top coded as 85 years of age or older and excluded since actual age was needed for accurate survival analysis. The final sample for analysis was 137,291.

Measures

Dependent Variable

Mortality was indicated as presumed yes or no as of December 31, 2019, based on CDC’s match with the National Death Index. Month and year of death were also provided for decedents.

Independent Variable

Measurement of spiritual practice as a complementary or alternative health practice varied between survey years, as seen in Table 1, although the response options remained constant. For this study, the created reference category of no included all the "no" responses during the year(s) each spiritual practice was asked in addition to all cases during years in which the spiritual practice question was not asked. Although prayer itself has many facets [18], we assumed that engaging in prayer or other spiritual practices when a person has health concerns is likely indicative of a longer-standing faith belief; and that established religious faith is often associated with a healthier lifestyle [19].

**Table 1:** Survey questions regarding spiritual practice for one's health in the National Health Interview Survey

Survey year(s)	Survey question	Responses
1999	People may also use alternative health care services. I'd like to ask you about your use of some alternative kinds of therapies and treatments. During the PAST 12 MONTHS have you used...?.spiritual healing/prayer	Yes, No, Refused, Don't know
2002, 2007	DURING THE PAST 12 MONTHS, did you pray specifically for the purpose of your OWN health?	Yes, No, Refused, Don't know
2012, 2017	DURING THE PAST 12 MONTHS, did you use ? Spiritual meditation including Centering Prayer and Contemplative Meditation?	Yes, No, Refused, Don't know

Covariates

Independent variables were selected to reflect the elements of the Andersen Behavioral Model of Health Services Utilization. This model offers a framework for examining how context and individual characteristics (i.e., predisposing, enabling, and need) influence health behaviors, including healthcare services, and health status [20].

Context

Indicated by survey year and four national Census regions.

Individual Predisposing

Defined by the CDC included self-identified sex (i.e., male, female); age at time of survey; and ethnicity (Hispanic yes/no). Race, education, and marital status were recoded by the authors as shown in Table 2.

**Table 2:** Survey-weighted characteristics of adults based on mortality status CI: confidence interval, HS: high school. Fisher exact p-values determined with Monte Carlo estimation of summarized, non-survey-weighted data. *For some variables, SAS software (version 9.4; SAS Institute Inc. Cary, NC, USA) was not able to calculate beyond zero.

	Died	Not died		
Survey n=	19,077	118,214	Rao-Scott	Fisher
Estimated annual population N=	25,086,559	192,808,997	Chi-square	p-value
	Percent (95% CI)	Percent (95% CI)		
Spiritual practice			1,438.4	0.0000*
None	67.9 (67.0-68.8)	78.8 (78.4-79.2)		
Spiritual healing	4.5 (4.1-4.9)	2.2 (2.1-2.3)		
Prayer for one's health	29.9 (26.1-27.8)	15.7 (15.4-16.1)		
Spiritual meditation	0.7 (0.5-0.8)	3.3 (3.1-3.5)		
Survey year			5,229.3	0.0000*
1999	34.7 (33.8-35.5)	15.9 (15.5-16.2)		
2002	29.4 (28.6-30.2)	17.2 (16.8-17.6)		
2007	20.2 (19.2-21.1)	20.1 (19.6-20.6)		
2012	12.2 (11.6-12.8)	22.3 (21.9-22.7)		
2017	3.6 (3.2-4.0)	24.6 (23.8-25.3)		
Census region			77.2	9.9×10^-45^
Northeast	17.8 (17.0-18.6)	18.3 (17.8-18.9)		
North Central/Midwest	24.8 (23.8-25.8)	23.6 (23.0-24.2)		
South	39.7 (38.6-40.9)	36.4 (35.7-37.1)		
West	17.7 (16.8-18.6)	21.7 (21.1-22.3)		
Individual pre-disposing				
Gender			84.3	2.6×10^-27^
Male	47.9 (47.1-48.7)	52.2 (51.8-52.6)		
Female	52.1 (51.3-52.9)	47.8 (47.4-48.2)		
Age at time of survey			16,347.8	0.0000*
18-44	11.6 (11.0,- 12.2)	55.8 (55.3-56.2)		
45-64	33.0 (32.2-33.9)	33.6 (33.2-33.9)		
65-84	55.4 (54.5-56.4)	10.7 (10.4-10.9)		
Race			202.7	1.2×10^-86^
White only	84.0 (83.3-84.8)	79.6 (79.1-80.1)		
Black/African American only	11.3 (10.7-12.0)	11.9 (11.5-12.3)		
Other and multiple race	4.6 (4.2-5.0)	8.4 (8.2-8.8)		
Ethnicity			532.3	3.4×10^-181^
Not Hispanic	93.6 (93.1-94.0)	85.7 (85.2-86.1)		
Hispanic	6.4 (6.0-6.9)	14.3 (13.9-14.8)		
Marital status			3,397.6	0.0000*
Married	55.7 (54.8-56.6)	56.1 (55.7-56.6)		
Widowed/Divorced/Separated	34.6 (33.7-35.4)	16.4 (16.2-16.7)		
Never married	9.7 (9.2-10.3)	27.5 (27.0-27.9)		
Education			2,261.9	0.0000*
Less than HS graduate	26.2 (25.4-27.0)	13.2 (12.9-13.5)		
High school graduate	34.8 (34.0-35.7)	27.3 (26.9-27.7)		
Some college, no 4-year degree	23.2 (22.5-23.9)	30.6 (30.1-31.0)		
Bachelor's degree or higher	15.8 (15.1-16.5)	29.0 (28.5-29.5)		
Individual pre-disposing				
Citizenship			648.5	2.6×10^-258^
US citizen	97.4 (97.2-97.7)	91.5 (91.2-91.8)		
Non-citizen	2.6 (2.3-2.8)	8.5 (8.2-8.8)		
Veteran			3,161.1	0.0000*
No	75.8 (75.1-76.6)	91.7 (91.5-91.9)		
Yes	24.2 (23.4-24.9)	8.3 (8.1-8.5)		
Poverty status			3.0	0.0857
At or above poverty level	89.1 (88.6-89.6)	89.6 (89.3-89.9)		
Below poverty level	10.9 (10.4-11.4)	10.4 (10.1-10.7)		
Uninsured			440.8	4.4×10^-253^
No	91.8 (91.3-92.3)	84.3 (84.0-84.6)		
Yes	8.2 (7.7-8.7)	15.7 (15.4-16.0)		
Individual need				
Self-reported health status			2,179.2	0.0000*
Excellent	12.2 (11.7-12.8)	32.1 (31.7-32.5)		
Very good to poor	87.8 (87.2-88.3)	67.9 (67.5-68.3)		
Psychological distress			153.0	1.7×10^-47^
No to low distress	86.3 (85.6-86.9)	89.6 (89.3-89.8)		
Moderate distress	9.2 (8.6-9.7)	7.7 (7.5-7.9)		
Serious distress	4.6 (4.2-5.0)	2.7 (2.6-2.8)		
Body mass index			18.6	2.8×10^-6^
Underweight to normal	39.0 (38.1-39.8)	40.8 (40.4-41.2)		
Overweight	34.0 (33.1-34.8)	33.6 (33.3-34.0)		
Obese	27.0 (26.3-27.9)	25.6 (25.2-25.9)		
Functional limitations			4,862.4	0.0000*
Limited in any way	59.1 (58.2-60.0)	28.2 (27.8-28.6)		
None or unknown	40.9 (40.0-41.8)	71.8 (71.4-72.2)		
Cancer			2,501.6	0.0000*
No	81.4 (80.8-82.1)	93.9 (93.7-94.1)		
Yes	18.6 (17.9-19.2)	6.1 (6.0-6.3)		
Coronary heart disease			3,449.1	0.0000*
No	86.6 (86.0-87.2)	97.4 (97.3-97.5)		
Yes	13.4 (12.8-14.0)	2.6 (2.5-2.7)		
Stroke			2,355.9	0.0000*
No	91.4 (90.9-91.9)	98.4 (98.3-98.5)		
Yes	8.6 (8.1-9.1)	1.6 (1.5-1.7)		
Heart attack			3,715.0	0.0000*
No	88.6 (88.1-89.1)	98.1 (98.0-98.2)		
Yes	11.4 (10.8-11.9)	1.9 (1.8-2.0)		
Diabetes			2,581.1	0.0000*
No/borderline	80.6 (79.9-81.2)	93.8 (93.7-93.9)		
Yes	19.4 (18.8-20.1)	6.2 (6.0-6.3)		
Health behavior				
Smoking status			2,418.3	0.0000*
Current smoker	25.0 (24.2-25.8)	18.9 (18.6-19.3)		
Former smoker	35.8 (35.0-36.6)	20.3 (19.9-20.6)		
Never smoked	39.2 (38.3-40.0)	60.8 (60.4-61.2)		
Any binge drinking past year			1,027.8	0.0000*
No	89.4 (88.9-89.0)	76.5 (76.1-77.0)		
Yes	10.6 (10.0-11.1)	23.5 (23.1-23.9)		
At least 10 minutes last week:				
Moderate exercise			1,139.5	0.0000*
No	60.9 (60.0-61.8)	43.8 (43.3-44.4)		
Yes	39.1 (38.2-40.0)	56.2 (55.6-56.7)		
Vigorous exercise			2,557.2	0.0000*
No	81.4 (80.6-82.1)	56.6 (56.1-57.1)		
Yes	18.6 (17.9-19.4)	43.8 (42.9-43.9)		
Strength training			693.7	0.0000*
No	88.9 (88.2-89.6)	76.5 (76.1-76.9)		
Yes	11.1 (10.4-11.8)	23.5 (23.1-23.9)		
Flu vaccination			1,706.2	0.0000*
Yes	50.6 (49.7-51.6)	26.9 (29.6-30.4)		
No	49.4 (48.4-50.3)	70.0 (69.6-70.4)		
Dentist visit			756.0	1.3×10^-251^
Yes	50.4 (49.5-51.3)	63.1 (62.6-65.5)		
No	49.6 (48.7-50.5)	36.9 (36.5-37.4)		

Individual enabling: Included federal poverty limit, US citizenship, veteran status, and health insurance (all yes/no).

Individual Health/Need

Included having any functional limitation (yes/no), self-reported health status (recoded by author to excellent versus all other response options), and body mass index (BMI) (recoded by author to underweight/normal (18.5-25), overweight (25-30), and obese (>30)). Specific medical conditions included having been diagnosed with cancer, coronary heart disease, stroke, heart attack, and diabetes (yes, no/borderline). Mental health was measured using the Kessler-6 scale, with findings from six questions summed and categorized into three groups of psychological distress (none to low, moderate, and serious) [21].

Health Behaviors

Self-reported health behaviors included smoking (measured as current, former, never smoked) and alcohol consumption (binge drinking episodes in the past year (numerical, recoded by author to yes/no)). Physical activity was assessed by the number of weekly sessions of 10 minutes (ranging from 0 to 28) of moderate and vigorous activity, as well as strength training. Following an approach used by prior NHIS researchers [22], we recoded these variables to (yes/no). Also, per Harrigan [22], we used yes/no for influenza vaccination in past year and recoded number of dentist visits in past year to yes/no.

Data management and analysis

There was modest missing data, with multiple possible codes. As noted in Table 1, "refused" and "don't know" were options for spiritual practice. The missing data options for most variables were "Unknown-refused", "Unknown-not ascertained", and "Unknown-don't know". For many variables, the numbers were small, such as 110 unknowns for cancer, up to 2,882 (2.1%) unknown number of 10-minute units of moderate exercise in the past week. For variables analyzed as “yes/no”, any response not coded as "yes" or "no" was recoded as a "no". For variables analyzed as more than two levels, we assigned missing cases to the reference category. For example, 2,196 (1.6%) subjects missing mental health status were coded as having “None to Low Psychological Distress”. We sought to avoid dropping subjects given the large number of covariates and relatively low number of subjects with spiritual practices and mortality in 2012 and 2017. In order to assess potential bias, we compared our cleaned dataset to a dataset where cases with any missing data were excluded. Findings are shown in Appendix 1. As seen, 20.3% of cases were excluded in the full drop file. Although there were minor differences in percentage point estimates, there were none to modest differences for most variables when comparing confidence intervals and no practical difference in basic regression findings. The confidence interval differences and difference percentage shown at bottom of Appendix 1 indicate the potential bias based on missing data for health behaviors. For our purposes, any group percentage differences in behavior based on spiritual practices should exceed those numbers, such as a 2.4 percentage point difference for moderate exercise.

Statistical analyses were performed using survey modules of SAS software (version 9.4; SAS Institute Inc. Cary, NC, USA). All analyses accounted for the complex, multistage, stratified, and cluster-sampling design by using given sampling weights, strata, and primary sampling unit (PSU) numbers [23]. The CDC provided adult sample weights which were further adjusted to account for those sample adults who could not be linked to the National Death Index.

First, we compared all characteristics of respondents based on mortality status, with bivariate testing for statistical significance using the survey-weighted Rao-Scott Chi-square test. In cases where the adjusted p-value was determined to be less than 0.0001, we calculated a Fisher exact p-value using summarized, but not survey-weighted values via Monte Carlo estimation. Second, we examined health behaviors by the three different types of spiritual practice. As with the overall mortality comparisons, we used Monte Carlo estimation of Fisher exact p-values for cases where the Rao-Scott p-value was less than 0.0001. However, due to smaller sample size for the 1999 surveys, we were able to directly run Fisher exact tests on non-survey-weighted data.

Third, to investigate associations between spiritual practice and all-cause mortality, we used multivariable Cox proportional hazard regression, with mortality as the dependent variable, attained age as the time variable and left truncation by age at time of study entry. As with the bivariate analyses, exact p-values were calculated for the hazard ratio t-tests. Multicollinearity was assessed by testing the correlations between included variables. We checked Cox proportional assumptions using both Schoenfeld residuals and log-log plot approaches. Log-log plots indicated that chronic diseases violated the constant proportional hazard assumptions, i.e., likelihood increased with increasing age. Thus, in addition to dummy variables for chronic disease, we added an additional continuous variable: dummy variable for each chronic disease * (each person’s age - mean sample age per year grouping). The mean sample age for 1999 was 44.212 years, for 2002/2007 was 44.812 years, and for 2012/2017 was 46.235 years. Finally, following the main regression model run as a single fully saturated block, we did an interaction analysis. We reran the main model, adding interaction terms of spiritual practices (with reference category as a "0") * all other variables, also as a fully saturated block. All p-values were two-tailed, with statistical significance thresholds set at p < 0.05.

## Results

Table 2 addresses research question 1 (spiritual practices and mortality) by presenting descriptive statistics based on mortality. There were statistically significant differences for all independent variables, except for poverty status. Those reporting prayer or spiritual healing were more frequently found among those who died, with the exception of those reporting spiritual meditation in the most recent years. Increasing age and chronic disease were positively associated with mortality.

Table 3 addresses research question 2 (spiritual practices and health behaviors). As seen below, in 1999, an estimated 27.0 million adults (13.7% of adults that year) reported using spiritual healing/prayer for their own health; in 2002 and 2007, an estimated 92.6 million annually (43.9% of adults) prayed for their own health; and in 2012 and 2017, an estimated 16.2 million (6.9% of adults) reported using spiritual meditation for their own health. Those responding yes to any of the spiritual practice questions were less frequently active smokers and more frequently visited a dentist and had flu shots. Those who prayed for their own health were less frequently binge drank but also exercised less frequently. Those who used spiritual healing or meditation exercised more frequently. There was a secular trend, with both obesity and exercise increasing over time and smoking decreasing. When respondents were asked about spiritual meditation including centering prayer and contemplative meditation, there was no significant difference with regards to weight status and binge drinking.

**Table 3:** Survey-weighted percentages of health behaviors based on one's prayer/spirituality for one's health est. pop.: estimated population, CI: confidence interval. Test = Rao-Scott Chi square. p-value is based on Rao-Scott if greater than 0.0001. Otherwise, Fisher's exact p-value is shown. p = p-value, with significance level set at <0.05.

	Spiritual healing (1999)			Prayer for one's health (2002, 2007)			Spiritual meditation (2012, 2017)		
	No	Yes		No	Yes		No	Yes	
Est. pop.	169,401,523	26,985,766		118,239,497	92,627,379		219,461,177	16,217,194	
	Percent (95% CI)	Percent (95% CI)	Test/p	Percent (95% CI)	Percent (95% CI)	Test/p	Percent (95% CI)	Percent (95% CI)	Test/p
Body mass index			16.50/0.0003			162.07/1.3×10^-20^			0.20/0.9028
Normal	45.0 (44.2-45.8)	43.1 (41.3-44.9)		44.3 (43.6-45.1)	39.2 (38.4-40.0)		37.5 (36.9-38.1)	37.9 (36.0-39.8)	
Overweight	34.5 (33.8-35.2)	33.0 (31.2-34.8)		34.0 (33.3-34.7)	33.4 (32.6-34.2)		33.4 (32.8-33.9)	33.0 (31.2-34.8)	
Obese	20.5 (19.9-21.1)	23.9 (22.4-25.4)		21.6 (21.0-22.2)	27.4 (26.7-28.2)		29.1 (28.6-29.7)	29.1 (27.4-30.8)	
Smoking status			33.46/4.5×10^-182^			113.84/2.7×10^-277^			20.83/3.2×10^-146^
Current smoker	24.6 (23.9-25.3)	19.5 (18.0-21.0)		23.5 (22.8-24.2)	18.8 (18.1-19.5)		16.4 (16.0-16.8)	13.8 (12.4-15.1)	
Former smoker	22.7 (22.1-23.4)	23.7 (22.0-25.3)		20.7 (20.0-21.3)	23.4 (22.6-24.1)		21.7 (21.2-22.1)	24.9 (23.0-26.8)	
Never smoked	52.6 (51.8-53.4)	56.8 (54.9-58.7)		55.8 (55.0-56.6)	57.8 (56.9-68.7)		61.9 (61.3-62.6)	61.3 (59.2-63.5)	
Binge drinking			55.98/1.7×10^-123^			407.93/3.6×10^-155^			0.30/0.5805
No	78.7 (78.0-79.5)	85.3 (83.8-86.7)		75.6 (74.8-76.4)	85.0 (84.3-85.6)		75.8 (75.2-76.4)	76.3 (74.5-78.1)	
Yes	21.3 (20.5-22.0)	14.7 (13.3-16.2)		24.4 (23.6-25.2)	15.0 (14.4-15.7)		24.2 (23.6-24.8)	23.7 (21.9-25.5)	
Moderate exercise			136.31/2.3×10^-62^			2.11/0.1464			216.72/2.0×10^-111^
No	52.7 (51.7-53.6)	40.0 (38.0-42.0)		50.0 (49.0-51.0)	49.1 (48.2-50.1)		41.3 (40.4-42.1)	25.9 (24.0-27.8)	
Yes	47.3 (46.4-48.3)	60.0 (58.0-62.0)		50.0 (49.0-51.0)	50.9 (49.9-51.8)		58.7 (57.9-59.6)	74.1 (72.2-76.0)	
Vigorous exercise			44.85/4.4×10^-263^			68.74/3.8×10^-241^			125.41/4.2×10^-229^
No	64.0 (63.2-64.9)	57.4 (55.4-59.3)		61.1 (60.1-62.0)	65.9 (65.0-66.8)		55.4 (54.7-56.1)	44.0 (42.0-46.0)	
Yes	36.0 (35.1-36.8)	42.6 (40.7-44.6)		38.9 (38.0-39.9)	34.1 (33.2-35.0)		44.6 (43.9-45.3)	56.0 (54.0-57.9)	
Strength training			44.42/1.8×10^-92^			12.74/0.0004			150.31/4.9×10^-78^
No	82.4 (81.7-83.0)	76.9 (75.3-78.5)		79.2 (78.5-79.9)	81.0 (80.2-81.7)		75.4 (74.8-75.9)	64.4 (62.4-66.3)	
Yes	17.6 (17.0-18.3)	23.1 (21.5-24.7)		20.8 (20.1-21.5)	19.0 (18.3-19.8)		24.6 (24.1-25.2)	35.6 (33.7-37.7)	
Flu vaccine			34.90/8.1×10^-62^			285.45/2.7×10^-115^			9.80/0.0017
No	73.7 (73.0-74.4)	68.7 (67.1-70.4)		75.9 (75.3-76.6)	67.3 (66.4-68.1)		61.5 (60.9-62.1)	58.1 (56.0-60.2)	
Yes	26.3 (25.6-27.0)	31.3 (29.6-32.9)		24.1 (23.4-24.7)	32.7 (31.9-33.6)		38.5 (37.9-39.1)	41.9 (39.8-44.0)	
Dentist visit			32.13/2.5×10^-61^			0.01/0.9186			35.15/4.7×10^-83^
No	38.6 (37.8-39.3)	33.5 (31.8-35.2)		39.3 (38.5-40.1)	39.2 (38.4-40.0)		38.2 (37.6-38.8)	32.5 (30.8-34.3)	
Yes	61.4 (60.7-62.2)	66.5 (64.8-68.2)		60.7 (59.9-61.5)	60.8 (60.0-61.6)		61.8 (61.2-62.4)	67.5 (65.7-69.2)	

Table 4 continues with research question 1 (spiritual practices and mortality) by presenting findings from a multivariable Cox regression model with mortality as the outcome. Of three spiritual practices measures, only prayer for your health was statistically significant: hazard ratio of 1.07 (95% confidence interval: 1.01-1.13, p=0.0124). Significant predisposing risk factors (p<0.001) included male sex (HR=1.51, 95% CI: 1.44-1.58) and unmarried status (HR=1.46, 95% CI: 1.36-1.56). The health status measures of specific diseases all had significantly higher hazards ratios, such as diabetes (HR=2.35, 95% CI: 2.13-2.60), while most healthy behaviors had reduced hazard ratios, such as moderate exercise (HR=0.86, 95% CI: 0.82-0.89).

**Table 4:** Proportional hazards (Cox) regression for mortality CI: confidence interval, ref.: reference, HS: high school. Statistical significance for individual HR determined by t-statistic test, with significance p<0.05.

	Hazard ratio	95% CI	t-statistic	p-value
Prayer/spirituality for own health				
No (ref.)	-	-	-	-
Spiritual healing	0.93	(0.86-1.00)	-1.96	0.0505
Prayer	1.07	(1.01-1.13)	2.50	0.0124
Spiritual meditation	1.05	(0.83-1.32)	0.37	0.7093
Survey year				
1999 (ref.)	-	-	-	-
2002	0.90	(0.86-0.94)	-4.25	2.32×10^-5^
2007	0.82	(0.78-0.88)	-6.26	5.49×10^-10^
2012	0.87	(0.81-0.92)	-4.50	7.40×10^-6^
2017	0.79	(0.70-0.89)	-3.87	1.16×10^-4^
Census region				
Northeast	0.87	(0.82-0.92)	-4.90	1.09×10^-6^
North Central/Midwest	0.94	(0.90-0.99)	-2.50	0.0124
South (ref.)	-	-	-	-
West	0.95	(0.90-0.99)	-2.17	0.0306
Individual pre-disposing				
Gender				
Male	1.51	(1.44-1.58)	17.61	1.88×10^-61^
Female (ref.)	-	-	-	-
Race				
White only (ref.)	-	-	-	-
Black/African American only	0.97	(0.92-1.03)	-1.10	0.2734
Other and multiple race	0.85	(0.77-0.93)	-3.59	0.0004
Ethnicity				
Not Hispanic (ref.)	-	-	-	-
Hispanic	0.72	(0.67-0.78)	-8.45	8.76×10^-17^
Marital status				
Married (ref.)	-	-	-	-
Widowed/Divorced/Separated	1.18	(1.13-1.22)	7.87	8.20×10^-15^
Never married	1.46	(1.36-1.56)	10.82	4.99×10^-26^
Education				
Less than HS graduate	1.06	(1.01-1.12)	2.52	0.012
High school graduate (ref.)	-	-	-	-
Some college, no 4-year degree	0.97	(0.92-1.02)	-1.17	0.2422
Bachelor's degree or higher	0.82	(0.78-0.87)	-7.03	3.53×10^-12^
Individual pre-disposing				
Citizenship				
US citizen (ref.)	-	-	-	-
Non-citizen	0.73	(0.65-0.82)	-5.25	1.86×10^-7^
Veteran				
No (ref.)	-	-	-	-
Yes	1.00	(0.95-1.05)	-0.16	0.8745
Poverty status				
At or above poverty level (ref.)	-	-	-	-
Below poverty level	1.11	(1.04-1.18)	3.41	0.0007
Uninsured				
No (ref.)	-	-	-	-
Yes	1.07	(0.99-1.14)	1.73	0.0838
Individual need				
Self-reported health status				
Excellent	0.75	(0.71-0.79)	-10.38	3.49×10^-24^
Very good to poor (ref.)	-	-	-	-
Psychological distress				
No to low distress (ref.)	-	-	-	-
Moderate distress	1.09	(1.02-1.17)	2.71	0.0069
Serious distress	1.25	(1.14-1.38)	4.67	3.32×10^-6^
Body mass index				
Underweight to normal (ref.)	-	-	-	-
Overweight	0.84	(0.80-0.87)	-8.10	1.47×10^-15^
Obese	0.94	(0.90-0.99)	-2.42	0.0157
Functional limitations				
Limited in any way	1.30	(1.25-1.36)	13.45	2.49×10^-38^
None or unknown (ref.)	-	-	-	-
Cancer				
No (ref.)	-	-	-	-
Yes	1.84	(1.66-2.05)	11.37	2.09×10^-28^
Coronary heart disease				
No (ref.)	-	-	-	-
Yes	1.47	(1.26-1.73)	4.77	2.07×10^-6^
Stroke				
No (ref.)	-	-	-	-
Yes	1.71	(1.42-2.05)	5.67	1.78×10^-8^
Heart attack				
No (ref.)	-	-	-	-
Yes	1.73	(1.45-2.03)	6.59	6.88×10^-11^
Diabetes				
No/borderline (ref.)	-	-	-	-
Yes	2.35	(2.13-2.60)	16.88	3.93×10^-57^
Cancer * age difference	0.98	(0.98-0.98)	-8.24	4.87×10^-16^
Coronary heart disease * age difference	0.99	(0.98-0.99)	-3.22	0.0013
Stroke * age difference	0.99	(0.98-0.99)	-2.29	0.0223
Heart attack * age difference	0.99	(0.98-0.99)	-3.93	9.14×10^-5^
Diabetes * age difference	0.98	(0.98-0.99)	-9.22	1.44×10^-19^
Health behavior				
Smoking status				
Current smoker	2.10	(2.00-2.20)	29.49	5.96×10^-142^
Former smoker	1.26	(1.20-1.31)	10.92	1.92×10^-26^
Never smoked (ref.)	-	-	-	-
Any binge drinking past year				
No (ref.)	-	-	-	-
Yes	0.96	(0.90-1.02)	-1.25	0.2124
At least 10 minutes in past week				
Moderate exercise				
No (ref.)	-	-	-	-
Yes	0.86	(0.82-0.89)	-7.96	4.25×10^-15^
Vigorous exercise				
No (ref.)	-	-	-	-
Yes	0.82	(0.80-0.86)	-8.21	5.93×10^-16^
Strength training				
Yes	0.99	(0.92-1.06)	-0.32	0.7477
No (ref.)	-	-	-	-
Flu vaccination				
Yes	1.05	(1.01-1.09)	2.30	0.0216
No (ref.)	-	-	-	-
Dentist visit				
Yes	0.84	(0.81-0.87)	-9.80	8.49×10^-22^
No (ref.)	-	-	-	-

Table 5 explores research question 3 (spiritual practices interactions) by presenting selected interaction terms from a model which included the regression model in Table 4 plus all interaction terms. The main effect of prayer for one’s health was no longer statistically significant after including interaction terms. Most of the significant interactions involved prayer for one’s health, and not the other measures of meditation or spiritual healing. With so many variables and interactions, it is possible to obtain spurious findings, the positive finding of strength training is likely an example. More plausibly, those who prayed and binge drank were more likely to die (HR=1.21, 95% CI: 1.02-1.44, p= 0.0255), even though binge drinking by itself was not significant in the main model. Men who used spiritual meditation including centering prayer and contemplative meditation were more likely to die (HR=2.03, 95% CI: 1.22-3.29, p=0.0068).

**Table 5:** Significant regression interaction terms based on Cox regression Significant regression interaction terms based on Cox regression from Table 4. Reference for interaction terms is no prayer/spirituality. HR: hazards ratio, CI: confidence interval, ref.: reference. Statistical significance for individual HR determined by t-statistic test, with significance p<0.05.

	Spiritual healing		Prayer for one's health		Spiritual meditation for health	
	HR (95% CI)	p-value	HR (95% CI)	p-value	HR (95% CI)	p-value
Main effect						
Spiritual practice						
None (ref.)	-		-		-	
Spiritual healing	0.81 (0.58-1.13)					
t-statistic	-1.24	0.2138				
Prayer for health	-		1.10 (0.94-1.29)		-	
t-statistic			1.23	0.219		
Meditation	-		-		0.90 (0.30-2.75)	
t-statistic					-0.18	0.8548
Selected interactions						
Sex						
Female (ref.)	-		-		-	
Male	1.07 (0.85-1.34)		0.93 (0.84-1.04)		2.03 (1.22-3.39)	
t-statistic	0.56	0.5753	-1.23	0.22	2.71	0.0068
Below poverty level						
No (ref.)	-		-		-	
Yes	1.05 (0.82-1.35)		1.15 (1.02-1.30)		0.92 (0.46-1.82)	
t-statistic	0.40	0.6928	2.26	0.024	-0.25	0.7993
Binge drinking						
No (ref.)	-		-		-	
Yes	1.00 (0.74-1.35)		1.21 (1.02-1.44)		1.31 (0.64-2.67)	
t-statistic	-0.01	0.9901	2.24	0.026	0.75	0.4536
Moderate exercise						
No (ref.)	-		-		-	
Yes	1.16 (0.98-1.38)		0.90 (0.82-0.98)		0.95 (0.57-1.59)	
t-statistic	1.75	0.0805	-2.4	0.017	-0.18	0.8539
Strength training						
No (ref.)	-		-		-	
Yes	0.93 (0.75-1.16)		1.23 (1.04-1.45)		0.80 (0.43-1.50)	
t-statistic	-0.62	0.5342	2.38	0.017	-0.69	0.4882

## Discussion

In this study of NHIS data from five annual surveys containing CAM supplements between 1999 and 2017, spiritual practices for health among adults were examined both as predictors of health-related behaviors during the survey year and for mortality during a follow-up period. Depending on how questions were worded during each survey year, the proportion of adults participating in spiritual practices for health per year varied substantially. There were also group differences in proportions of health behaviors such as exercise and binge drinking depending on how the spiritual practice question was asked. Such differences were detectable, even with secular changes in health behaviors over the study period, such as a slight increase in any annual binge drinking and a moderate increase in weekly exercise. Regardless of wording, the frequency of spiritual practice for one’s health was observed to be significantly higher among those who died. After adjusting for multiple adult characteristics such as age and health status, prayer for one’s health was still positively associated with mortality. However, that main effect was slight and disappeared when spiritual practices were interacted with other variables.

Many researchers studying various populations have found that age and religiosity, including frequency of prayer, are positively associated [1,2,11]. Indeed, religious beliefs and practices provide ways of coping with suffering and existential threat [24], so religious practices might be expected to increase with illness and impending death. Thus, it is surprising that spiritual practice for health was infrequent for some survey years.

The variation in frequence of spiritual practices observed during this study period, as well as its overall decline, may be explainable. The years when the frequency of spiritual practices observed was highest (i.e., 2002, 2007) were the years when the survey item inquired specifically did you pray. . .? During other years, the items used the vague and less traditional language of spiritual healing/prayer and spiritual meditation including centering prayer and contemplative meditation. It is likely that many Americans did not know what centering prayer or contemplation were; in 1999, meditation may have been perceived as spiritually avantgarde [25]. Concurrently, religious affiliation was decreasing during the study period. Although prayer is a spiritual practice that some nonaffiliated Americans do practice, a very slight downward trend in prayer frequency has occurred over the past few decades [1].

The positive interaction of prayer for one’s health and poverty is consistent with theories that religious belief is fueled by deprivation and disadvantage [1,25]. Regarding the interaction between prayer and binge drinking, the survey data does not address these qualitative elements, but other studies suggest that those who binge drink are more likely to be experiencing psychological despair or spiritual/existential struggle [26,27].

Limitations

Although there are many advantages to a large, population-based survey, there are also limitations. A major weakness of NHIS for population surveillance of spiritual practices is that specific questions could change each year, thus measuring different types or aspects of spiritual practice. With survey questions varying by year, there is a confluence of survey content and time which can be partially addressed by multiple regression, but not entirely resolved. This was partially addressed by comparing health behaviors by spiritual practice separately for the three time periods. There is a risk of misclassification due to handling of missing data, in our case recoding rather dropping. However, we were able to demonstrate that the actual impact is likely to be low for most variables, with the biggest impact being for strength training in past week. With these crude estimates, we have a threshold to decide how much of a group difference should be taken as "real". This topic also suffers from confounding by indication or reverse causality. One is most likely to undertake a spiritual practice for health because of poor health. With cross-sectional data, we cannot determine which came first, spirituality or poor health. And although we have many measures of health, they are limited and do not reflect the person's entire health status at time of survey. Also, with a large dataset and many variables, particularly interaction terms, there is a probability of Type I errors, i.e., statistically significant findings which are not really true. In this study, we primarily used common sense. For example, it seems unlikely that engaging in both spiritual practice and exercise would increase likelihood of mortality.

Another major limitation is the lack of additional spirituality data collected, such as subjective experience of individuals, perception of efficacy, or other beliefs (e.g., perceived lovingness of the God, found to explain frequency of healing prayer) [10] which leaves a large gap in understanding why and how Americans use spiritual practices for their health. One view is that religious belief influences health behavior to the extent with which individuals view their body as “sacred” [28]. Given that the dataset has no additional religious information, we could only examine potential correlation between health-related prayer and health-related behavior. Similarly, if there were formal religious measures, such as frequency of worship program attendance, one might decipher if individuals could be placed in a restful religious category (typically good health) or crisis religiousness/spiritual practice by oneself only when in trouble (generally poor health) [29].

Due to age top-coding in our study dataset, we were only able to examine up to 84 years of age using survival analysis. Some have found that religion is particularly more important among those with much higher age [30]. Another limitation is that it is unknown whether individuals experienced changes in spiritual practices or other independent variables in the years after completing the surveys. Although religiosity generally increases with age, there are multiple factors involved across a person’s lifespan [31]. Finally, since the close of this study period, there may be a stronger impact of spiritual practices due to the COVID-19 pandemic.

## Conclusions

This population-based survey demonstrated that in any given year, millions of American adults engaged in spiritual practices such as prayer or spiritual meditation for personal health. Engaging in these spiritual practices was positively associated with mortality in the years following the survey. However, that impact faded when considering socio-demographics, health measures, and health behaviors. Those engaging in spiritual practice for health were more likely to refrain from smoking and receive flu vaccinations. However, depending on the specific spiritual practice, there may not have been differences from other adults during that survey year in other health behaviors, such as exercise and binge drinking. Understanding of differing associations between spiritual practices and health behaviors may be helpful in improving health interventions.

## Appendices

Appendix 1

**Table 6:** Sensitivity analysis, population estimates, and survival analysis based on missing data Full cases as used in study, full drop is dropping cases with any missing data. CI: confidence interval, HR: hazard ratio, HS: high school. + CI difference: highest or lowest CI of full cases - highest or lowest CI of full drop, Percentage is CI difference/mean of full cases. *** p<0.0001.

	Full cases	Full drop			Full cases		Full drop	
Survey n=	137,291	109,335						
	Percent (95% CI)	Percent (95% CI)	CI difference+		HR (95% CI)	p-value	HR (95% CI)	p-value
Died	11.5 (11.3-11.8)	10.2 (10.0-10.5)	0.8	7.0%				
Spiritual practice								
None	77.5 (77.2-77.9)	77.6 (77.2-78.0)	0.0	0.0%	-		-	
Spiritual healing	2.5 (2.4-2.6)	2.4 (2.3-2.6)	0.0	0.0%	0.94 (0.87-1.01)	0.0814	0.97 (0.88-1.06)	0.4378
Prayer for one's health	17.0 (16.7-17.4)	16.6 (16.2-17.0)	0.0	0.0%	1.08 (1.03-1.14)	0.003	1.1 (1.03-1.18)	0.0032
Spiritual meditation	3.0 (2.8-3.1)	3.4 (3.2-3.6)	-0.1	-3.3%	1.05 (0.83-1.33)	0.6511	1.05 (0.82-1.35)	0.6934
Survey year								
1999	18.0 (17.7-18.4)	16.6 (16.2-17.0)	0.7	3.9%	-		-	
2002	18.6 (18.2-19.0)	16.7 (16.4-17.1)	1.1	5.9%	0.89 (0.85-0.94)	***	0.88 (0.82-0.93)	***
2007	20.1 (19.6-20.6)	20.2 (19.6-20.7)	0.0	0.0%	0.8 (0.75-0.85)	***	0.79 (0.73-0.86)	***
2012	21.1 (20.7-21.5)	22.6 (22.1-23.0)	-0.6	-2.8%	0.84 (0.79-0.90)	***	0.85 (0.79-0.91)	***
2017	22.1 (21.4-22.9)	23.9 (23.1-24.7)	-0.2	-0.9%	0.76 (0.67-0.85)	***	0.74 (0.65-0.85)	***
Census region								
Northeast	18.3 (17.8-18.8)	17.7 (17.1-18.2)	0.0	0.0%	0.94 (0.90-0.99)	0.0091	0.92 (0.87-0.98)	0.0045
North Central/Midwest	23.7 (23.2-24.3)	23.7 (23.1-24.3)	0.0	0.0%	0.87 (0.82-0.92)	***	0.89 (0.83-0.95)	***
South	36.8 (36.1-37.5)	37.0 (36.3-37.7)	0.0	0.0%	-		-	
West	21.2 (20.6-21.8)	21.7 (21.1-22.3)	0.0	0.0%	0.95 (0.90-1.00)	0.0326	0.94 (0.88-0.99)	0.0279
Individual pre-disposing								
Age at time of survey								
18-44	50.7 (50.3-51.1)	51.9 (51.5-52.3)	-0.4	-0.8%				
45-64	33.5 (33.2-33.8)	33.6 (33.3-34.0)	0.0	0.0%				
65-84	15.8 (15.5-16.1)	14.4 (14.2-14.7)	0.8	5.1%				
Male	48.3 (48.0-48.7)	49.2 (48.8-49.6)	-0.1	-0.2%	1.51 (1.44-1.58)	***	1.46 (1.39-1.54)	***
Hispanic	13.4 (13.0-13.9)	13.4 (12.9-13.8)	0.0	0.0%	0.72 (0.67-0.78)	***	0.72 (0.66-0.79)	***
Race								
White only	80.1 (79.7-80.6)	80.3 (79.9-80.5)	0.0	0.0%	-		-	
Black/African American only	11.9 (11.5-12.2)	11.6 (11.2-12.0)	0.0	0.0%	0.97 (0.91-1.02)	0.252	1.02 (0.95-1.08)	0.6005
Other and multiple race	8.0 (7.7-8.3)	8.1 (7.8-8.4)	0.0	0.0%	0.86 (0.79-0.95)	0.001	0.86 (0.77-0.95)	0.0036
Marital status								
Married	56.1 (55.6-56.5)	56.4 (56.0-56.9)	0.0	0.0%	-		-	
Widowed/Divorced/Separated	18.5 (18.2-18.8)	18.1 (17.8-18.4)	0.0	0.0%	1.17 (1.12-1.22)	***	1.14 (1.09-1.19)	***
Never married	25.4 (25.0-25.8)	25.5 (25.0-25.9)	0.0	0.0%	1.45 (1.36-1.56)	***	1.43 (1.32-1.55)	***
Education								
Less than HS graduate	14.7 (14.4-15.0)	13.8 (13.5-14.2)	0.2	1.4%	1.05 (1.00-1.11)	0.0549	1.04 (0.98-1.10)	0.1803
High school graduate	28.2 (27.8-28.5)	26.5 (26.1-26.9)	0.9	3.2%	-		-	
Some college, no 4-year degree	29.7 (29.3-30.1)	30.5 (30.1-30.9)	0.0	0.0%	0.97 (0.92-1.02)	0.4283	0.98 (0.92-1.03)	0.4122
Bachelor's degree or higher	27.4 (27.0-28.0)	29.1 (28.6-29.6)	-0.6	-2.2%	0.82 (0.78-0.87)	***	0.85 (0.80-0.90)	***
Individual pre-disposing								
Non-US Citizen	8.0 (7.6-8.0)	7.5 (7.2-7.7)	0.0	0.0%	0.72 (0.64-0.81)	***	0.74 (0.64-0.85)	***
Veteran	10.1 (9.9-10.3)	10.2 (9.9-10.4)	0.0	0.0%	0.99 (0.94-1.04)	0.5467	0.98 (0.92-1.04)	0.4732
Below poverty level	10.4 (10.2-10.8)	11.6 (11.3-12.0)	-0.5	-4.8%	1.13 (1.06-1.20)	***	1.13 (1.06-1.21)	***
Uninsured	14.8 (14.6-15.1)	14.5 (14.2-14.8)	0.0	0.0%	1.06 (0.99-1.14)	0.1158	1.05 (0.97-1.15)	0.2127
Individual need								
Excellent self-reported health	29.8 (29.4-30.1)	30.6 (30.2-31.0)	0.0	0.0%	0.74 (0.70-0.78)	***	0.73 (0.68-0.77)	***
Psychological distress								
No to low distress	89.2 (88.9-89.4)	88.8 (88.6-89.1)	0.0	0.0%	-		-	
Moderate distress	7.9 (7.7, 8-.1)	8.2 (8.0-8.4)	0.0	0.0%	1.11 (1.04-1.18)	0.0041	1.11 (1.04-1.20)	0.0033
Serious distress	2.9 (2.8-3.0)	3.0 (2.8-3.1)	0.0	0.0%	1.31 (1.19-1.44)	0.0008	1.33 (1.19-1.48)	***
Body mass index								
Underweight to normal	40.6 (40.2-40.9)	37.7 (37.3-38.1)	2.1	5.2%	-		---	
Overweight	33.7 (33.4-34.0)	35.0 (34.6-35.3)	-0.6	-1.8%	0.84 (0.80-0.88)	***	0.81 (0.77-0.86)	***
Obese	25.7 (25.4-26.1)	27.3 (26.9-27.7)	-0.8	-3.1%	0.96 (0.92-1.01)	0.0069	0.94 (0.89-0.99)	0.0199
Any functional limitations	31.7 (31.4-32.1)	31.8 (31.3-32.2)	0.0	0.0%	1.31 (1.26-1.36)	***	1.3 (1.24-1.37)	***
Any cancer	7.6 (7.4-7.7)	7.6 (7.4-7.8)	0.0	0.0%	1.23 (1.17-1.29)	***	1.26 (1.20-1.34)	***
Any coronary heart disease	3.8 (3.7-4.0)	3.8 (3.6-3.9)	0.0	0.0%	1.14 (1.07-1.21)	***	1.16 (1.08-1.25)	***
Any stroke	2.4 (2.3-2.5)	2.3 (2.2-2.4)	0.0	0.0%	1.26 (1.18-1.35)	***	1.27 (1.18-1.38)	***
Any heart attack	3.0 (2.9-3.1)	2.8 (2.7-3.0)	0.0	0.0%	1.39 (1.30-1.48)	***	1.41 (1.31-1.52)	***
Any diabetes	7.7 (7.5-7.9)	7.5 (7.3-7.7)	0.0	0.0%	1.58 (1.51-1.66)	***	1.64 (1.56-1.73)	***
Health behavior								
Smoking status								
Current smoker	19.6 (19.3-20.0)	19.7 (19.4-20.1)	0.0	0.0%	2.14 (2.03-2.24)	***	2.18 (2.05-2.32)	***
Former smoker	22.1 (21.7-22.4)	22.2 (21.8-22.5)	0.0	0.0%	1.26 (1.21-1.31)	***	1.25 (1.19-1.32)	***
Never smoked	58.3 (57.9-58.7)	58.1 (57.6-58.5)	0.0	0.0%	-		-	
Any binge drinking past year	22.0 (21.6-22.4)	24.1 (23.7-24.5)	-1.3	-5.9%	0.95 (0.90-1.01)	0.2912	0.97 (0.91-1.03)	0.3214
Any moderate exercise	54.2 (53.7-54.7)	57.7 (57.1-58.3)	-2.4	-4.4%	0.86 (0.83-0.90)	***	0.86 (0.82-0.90)	***
Vigorous exercise	40.5 (40.0-41.0)	42.8 (42.2-43.3)	-1.2	-3.0%	0.82 (0.78-0.86)	***	0.82 (0.77-0.87)	***
Any strength training	22.1 (21.7-22.4)	27.1 (26.7-27.5)	-4.3	-19.5%	0.99 (0.92-1.06)	***	0.99 (0.92-1.07)	0.7819
Any flu vaccination	32.4 (32.0-32.8)	33.1 (32.7-33.5)	0.0	0.0%	1.05 (1.01-1.09)	0.0188	1.03 (0.98-1.08)	0.2753
Any dentist visit	61.6 (61.2-62.0)	63.1 (62.6-63.5)	-0.6	-1.0%	0.84 (0.81-0.87)	***	0.83 (0.80-0.87)	***
